# The large bilateral gemination of permanent maxillary central incisors: A rare developmental dental anomaly case report

**DOI:** 10.1016/j.radcr.2026.01.015

**Published:** 2026-02-05

**Authors:** Bita Heydarzadeh, Saba Khorram, Fereshteh Hayatimotlagh

**Affiliations:** Department of Oral and Maxillofacial Radiology, School of Dentistiry, Shahid Beheshti University of Medical Sciences, Tehran Province, Velenjak, Daneshjoo Blvd, School of Dentistiry, 1983969411 Tehran, Iran

**Keywords:** Cone-beam computed tomography, Dental anomaly, Gemination

## Abstract

Bilateral gemination of permanent maxillary central incisors is a rare developmental dental anomaly, with a prevalence of approximately 0.04% in permanent dentition. This case report describes a 12-year-old boy presenting with enlarged central incisors, featuring incisal notches and causing palatal displacement of adjacent lateral incisors due to space encroachment. Cone-beam computed tomography (CBCT) revealed 2 distinct root canals with divergent apices in the right incisor and a single bean-shaped canal in the left, with no periapical pathology. These findings highlight CBCT’s critical role in mapping complex pulpal anatomy for accurate diagnosis. The condition predisposes teeth to plaque retention, increasing risks of caries and gingival inflammation. Management requires a multidisciplinary approach, including conservative enameloplasty, restorative procedures, or orthodontic intervention to address aesthetic and functional challenges. Further research is needed to refine treatment protocols and explore the etiology of such anomalies.

## Introduction

Gemination is a developmental dental anomaly characterized by the incomplete division of a single tooth germ, resulting in a tooth with a bifid or enlarged crown, typically sharing a single root and pulp chamber [1,2]. This condition differs from fusion, where 2 separate tooth buds unite, often leading to a single large tooth with potentially separate roots and a reduced total tooth count in the arch [3,4]. Gemination most commonly affects the anterior region, particularly the maxillary incisors, and while it usually presents unilaterally, bilateral cases are exceptionally rare [5,6].

The prevalence of gemination varies by dentition and population. In the permanent dentition, it occurs in approximately 0.1% to 0.5% of cases, with bilateral involvement reported at rates as low as 0.04% [7,8]. It is more frequent in the primary dentition, with estimates ranging from 0.5% to 0.7%, and may sometimes affect successor permanent teeth [9]. Etiological factors include genetic influences, environmental factors such as trauma during odontogenesis, nutritional deficiencies, or systemic conditions [1,10]. There is no consistent gender predilection, though some studies indicate a slight male predominance in specific populations [7].

Clinically, geminated teeth may present aesthetic concerns, malocclusion, or functional issues, particularly in the anterior zone. The cleft or groove in the crown can promote plaque retention, increasing risks of caries, periodontal disease, and potential pulpal involvement [4,8]. Management options include conservative monitoring, restorative procedures, orthodontic alignment, or surgical interventions like hemisection, tailored to the case's severity [4,5].

This case report details a rare instance of bilateral gemination in the maxillary central incisors of a young patient, emphasizing diagnostic challenges and treatment outcomes to add to the sparse literature on bilateral presentations [2,6].

## Case report

A 12-year-old boy was referred to the oral and maxillofacial radiology department for the cone-beam computed tomography (CBCT) evaluation of maxillary teeth number 1, 2, 3, right and left. The chief complaint includes dental malalignment and enlarged maxillary anterior teeth. There was neither a remarkable medical nor a family history of dental anomalies.

**Fig. 1:**
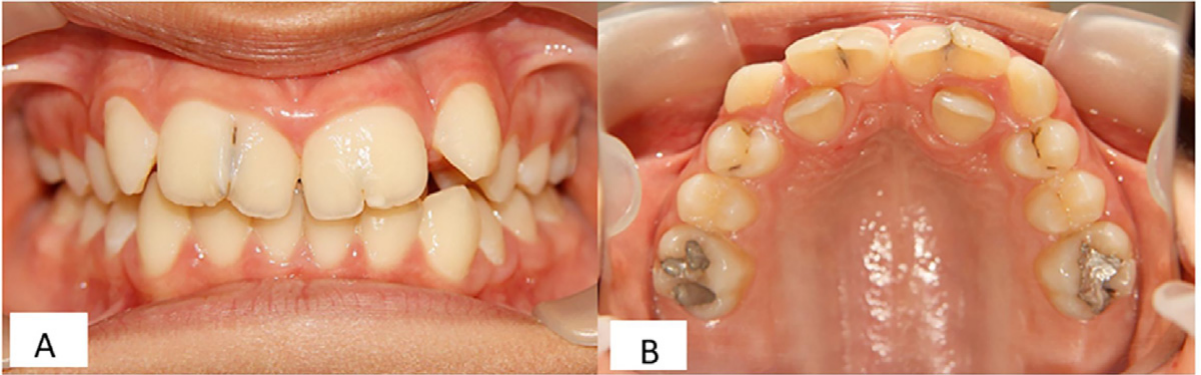
Clinical view of bilateral geminated permanent maxillary central incisors with notches. (A) Frontal view and (B) Palatal view.

Intraoral examination revealed permanent central maxillary incisors, with the width of 12.4 mm in the right central incisor and 11.8 mm in the left central incisor, which had incisal notches, with depth of 1.2 mm and 0.9 mm in the right and left maxillary incisors, respectively. Thermal pulp testing, percussion and periodontal probing showed no abnormalities. The number of teeth was normal. The patient reported no functional problems (e.g., speech or chewing difficulties), but the aesthetic appearance of the enlarged and notched anterior teeth was a primary concern for the adolescent patient and his family. The space between primary maxillary left and right lateral incisors had been completely filled by enlarged teeth so that both lateral incisors were also palatally displaced (Fig. 1).

CBCT was chosen over conventional 2D radiographs because of its superior ability to provide 3-dimensional visualization of the complex and asymmetric root canal anatomy in geminated teeth, which is often obscured by superimposition in periapical or panoramic images.

**Fig. 2:**
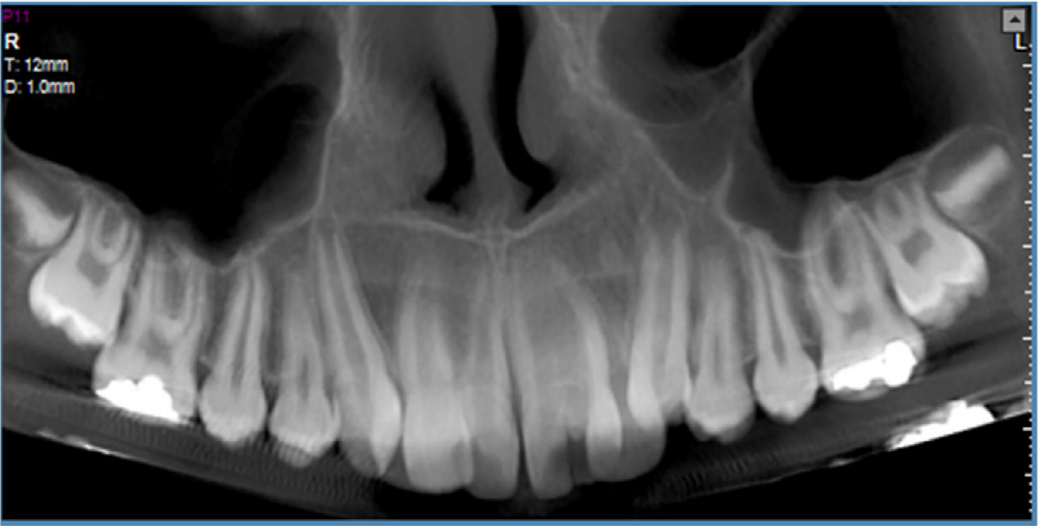
Reformatted panoramic.

**Fig. 3:**
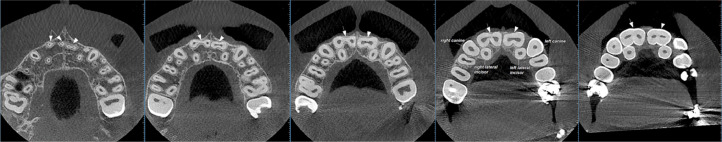
Axial view of CBCT images. From left to right, the images are arranged from the apex to the crown of the teeth, showing the pathway and connectivity of the root canals in the right (arrow) and left (arrowhead) geminated maxillary incisors.

**Fig. 4:**
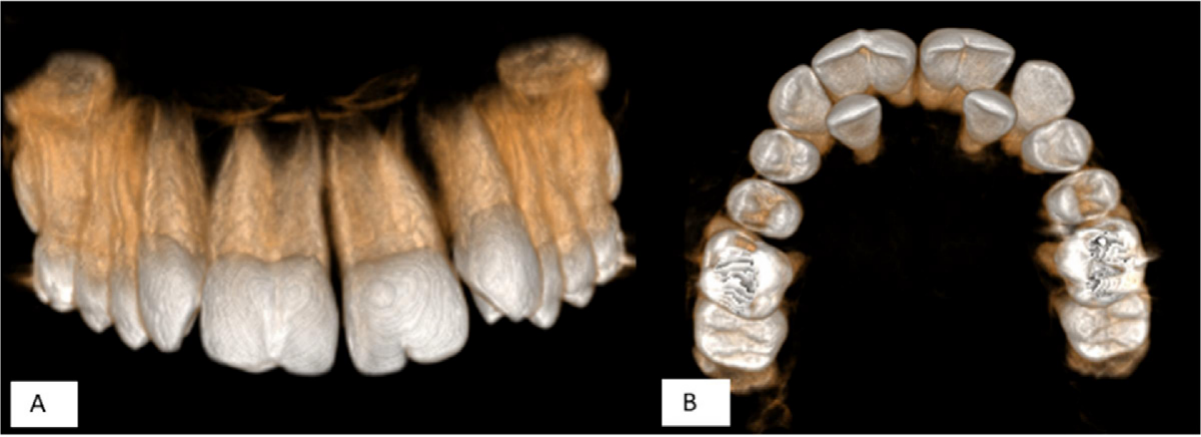
Three-dimensional reconstructed view. (A) Anterior view and (B) Palatal view.

The CBCT imaging obtained from the patient's maxilla revealed the following findings: The right central incisor exhibited 2 distinct canals originating from the pulp chamber and extending to the apex. The roots of this tooth diverged at the apical end. The left central incisor presented a single root with a single, bean-shaped canal. No periapical lesions, inflammation, or bone resorption were observed around the teeth mentioned above. The lateral incisors had erupted palatally due to insufficient space, while the remaining teeth displayed normal position and morphology (Figs. 2, Fig. 3, Fig. 4).

Based on clinical examinations and imaging, the large bilateral gemination of permanent maxillary central incisors was diagnosed. The proposed treatment plan including preventive measures, sealants for grooves, conservative enameloplasty, composite restorations for aesthetics and plaque control, orthodontic alignment for malocclusion, or more complex interventions (eg, endodontic therapy followed by hemisection or prosthetic rehabilitation with veneers/crowns was explained; unfortunately, our patient’s parents could not afford any treatment plan. Ulimately, the patient's parents were emphasized that in the absence of treatment, there is a likelihood of progression the risk of caries, periodontal disease, malocclusion and possible pulpal involvement over time.

## Discussion

Bilateral gemination of the permanent maxillary central incisors, as observed in this 12-year-old patient, is an exceptionally rare developmental dental anomaly, with a reported prevalence of approximately 0.04% in permanent dentition [7]. The clinical presentation, characterized by enlarged crowns (12.4 mm and 11.8 mm for the right and left incisors, respectively) with incisal notches and distinct root canal morphologies—two separate canals in the right incisor versus a single bean-shaped canal in the left—highlights the diagnostic complexity of this condition [11]. Such bilateral involvement in the anterior maxilla is infrequently reported, making this case a significant contribution to the existing literature [5,12].

The use of CBCT was justified in this 12-year-old patient due to the diagnostic challenge posed by the asymmetric root canal configurations and potential partial root divergence, features that cannot be reliably assessed with conventional 2D imaging. This indication aligns with current evidence-based guidelines for developmental dental anomalies. Radiation exposure was minimized according to the ALARA principle by employing a small field of view limited to the anterior maxilla and age-optimized low-dose protocols. CBCT was pivotal in delineating the divergent root apices of the right incisor and the unique canal configuration of the left, corroborating studies that emphasize CBCT’s role in mapping complex pulpal anatomy for accurate diagnosis and treatment planning [13]. The absence of periapical pathology or periodontal complications at presentation suggests early detection, yet the incisal notches and enlarged crowns predispose the teeth to plaque retention, increasing the risk of caries or gingival inflammation if untreated [4]. The palatal displacement of the lateral incisors due to space encroachment by the geminated teeth further underscores the functional and aesthetic challenges, consistent with reports linking anterior gemination to malocclusion and crowding [3].

The etiology of gemination is multifaceted, likely involving genetic predispositions and environmental factors such as microtrauma or nutritional deficiencies during odontogenesis [10]. The lack of familial history in this case suggests a sporadic occurrence, a pattern noted in nonsyndromic dental anomalies [14].

Management of bilateral gemination requires a tailored, multidisciplinary approach. Conservative strategies, such as enameloplasty to seal grooves or composite restorations for aesthetic improvement, are often preferred for asymptomatic cases [4]. In cases with significant malocclusion, orthodontic intervention or, in complex scenarios, surgical options like hemisection may be considered [5,6]. Financial constraints in this case precluded treatment, highlighting socioeconomic barriers to pediatric dental care [15]. Future management could involve preventive sealants and periodic CBCT monitoring, with aesthetic solutions like veneers considered at skeletal maturity [12].

The inability to afford even basic interventions in this case highlights a common socioeconomic barrier in pediatric dental care [15]. Although no treatment was performed, emphasis was placed on accessible preventive measures and a recommended follow-up strategy to mitigate long-term risks. This real-world scenario underscores the need for early, low-cost preventive approaches in resource-limited settings when managing rare developmental anomalies.

Unlike most reported bilateral cases, which typically present symmetric root canal anatomies [5,6], this case demonstrates asymmetry, with the right incisor showing 2 distinct canals and divergent apices—potentially indicating partial root separation—versus the left’s single bean-shaped canal. This variability highlights the unpredictable nature of gemination and underscores the value of CBCT in revealing such nuances [13]. The crown sizes (12.4 mm and 11.8 mm) are among the largest reported, surpassing many cases but comparable to the 13 mm and 12 mm in Shokri et al. [5], further exacerbating the malocclusion. The asymmetric canal configuration documented here is a novel addition, as most literature describes symmetric bilateral geminations [5,6,11]. This enriches the understanding of morphological variability and advocates for advanced imaging in similar cases.

This case enriches the limited literature on bilateral maxillary gemination by documenting its morphological variability and clinical implications. It underscores the critical role of advanced imaging in diagnosis and advocates for individualized treatment plans to address both functional and aesthetic concerns. Longitudinal studies are recommended to refine management protocols and explore the genetic underpinnings of such anomalies.

## Patient consent

The patient provided informed consent for his clinical information and images to be included in this case report. He understood the purpose of the publication, how his privacy would be protected, and agreed voluntarily to share his data for research purposes.
